# Sigma-1 Receptor Agonists Directly Inhibit Na_V_1.2/1.4 Channels

**DOI:** 10.1371/journal.pone.0049384

**Published:** 2012-11-05

**Authors:** Xiao-Fei Gao, Jin-Jing Yao, Yan-Lin He, Changlong Hu, Yan-Ai Mei

**Affiliations:** School of Life Sciences, Institutes of Brain Science and State Key Laboratory of Medical Neurobiology, Fudan University, Shanghai, China; Indiana University School of Medicine, United States of America

## Abstract

(+)-SKF 10047 (N-allyl-normetazocine) is a prototypic and specific sigma-1 receptor agonist that has been used extensively to study the function of sigma-1 receptors. (+)-SKF 10047 inhibits K^+^, Na^+^ and Ca2+ channels via sigma-1 receptor activation. We found that (+)-SKF 10047 inhibited Na_V_1.2 and Na_V_1.4 channels independently of sigma-1 receptor activation. (+)-SKF 10047 equally inhibited Na_V_1.2/1.4 channel currents in HEK293T cells with abundant sigma-1 receptor expression and in COS-7 cells, which barely express sigma-1 receptors. The sigma-1 receptor antagonists BD 1063,BD 1047 and NE-100 did not block the inhibitory effects of (+)-SKF-10047. Blocking of the PKA, PKC and G-protein pathways did not affect (+)-SKF 10047 inhibition of Na_V_1.2 channel currents. The sigma-1 receptor agonists Dextromethorphan (DM) and1,3-di-o-tolyl-guanidine (DTG) also inhibited Na_V_1.2 currents through a sigma-1 receptor-independent pathway. The (+)-SKF 10047 inhibition of Na_V_1.2 currents was use- and frequency-dependent. Point mutations demonstrated the importance of Phe^1764^ and Tyr^1771^ in the IV-segment 6 domain of the Na_V_1.2 channel and Phe^1579^ in the Na_V_1.4 channel for (+)-SKF 10047 inhibition. In conclusion, our results suggest that sigma-1 receptor agonists directly inhibit Na_V_1.2/1.4 channels and that these interactions should be given special attention for future sigma-1 receptor function studies.

## Introduction

The sigma receptor was originally described as a novel opioid receptor subtype, but it is now considered to be a unique receptor [1], [2]. Sigma receptors consist of two subtypes: sigma-1 and sigma-2. The sigma-1 receptor was first cloned from guinea pigs in 1996 [3], [4], [5], but the sigma-2 receptor has not been cloned. The sigma-1 receptor is widely expressed in the brain and peripheral organs, and it may be involved in numerous processes, such as Alzheimer's disease, schizophrenia, pain, drug addiction, stroke, cancer, depression and anxiety [2], [6]. The molecular mechanisms of sigma-1 receptor effects in these diseases are not understood. One of the most important molecular actions of sigma-1 receptors is the modulation of various voltage- and ligand-gated ion channels [2], [7], [8].

Voltage-gated sodium channels initiate and propagate action potentials in excitable cells. Nine voltage-gated sodium channel isoforms have been identified in mammals [9], [10]. Na_V_1.2 is the most abundant sodium channel isoform in the central nervous system comprising approximately 80% of the total rat brain voltage-gated sodium channels [11]–[12], and it controls axonal action potential conduction and neurotransmitter release in presynaptic terminals [13]. Na_V_1.2 mutations cause inherited febrile seizures and epilepsy [9]. The Na_V_1.4 channel is the predominant voltage-gated Na^+^ channel isoform in skeletal muscle [14], and various channel mutations are associated with muscular diseases, including potassium-aggravated myotonia, paramyotonia congenita, hyperkalemic periodic paralysis, hypokalemic periodic paralysis and normokalemic periodic paralysis [15]. The major cardiac voltage-gated Na^+^ channel is Na_V_1.5 [16], [17], which is involved in many arrhythmic disorders, such as long-QT syndrome type 3, Brugada syndrome, conduction disease, sinus node dysfunction and atrial standstill [18], [19].

(+)-SKF 10047 is a prototypic and specific sigma-1 receptor agonist that has been extensively used to investigate sigma-1 receptor function. (+)-SKF 10047 inhibits cardiac Na_V_1.5 channels in HEK293 cells, COS-7 cells and cardiac myocytes [20], [21], but little is known about Na_V_1.2/Na_V_1.4 modulation by sigma-1 receptor activation.

We found that (+)-SKF 10047 inhibited Na_V_1.2 and Na_V_1.4 channel currents, but these inhibitory effects were independent of sigma-1 receptor activation. (+)-SKF 10047 inhibited Na_V_1.2/1.4 channel currents equally in HEK293T cells (which have abundant sigma-1 receptor expression) and COS-7 cells (which barely express sigma-1 receptors). The present study is the first report of the direct Na_V_1.2/1.4 channel current inhibition by sigma-1 receptor agonists, which should be given special attention for investigation of sigma-1 receptor function.

## Materials and Methods

### Ethics statement

This study was carried out in strict accordance with the recommendations in the Guide for the Care and Use of Laboratory Animals of the National Institutes of Health. The protocol was approved by the Committee on the Ethics of Animal Experiments of the Fudan University (Permit Number: 2007-0002). All surgery was performed under sodium pentobarbital anesthesia, and all efforts were made to minimize suffering.

### Chemicals

H-89, PKAI, BIM I, GTPγS, lidocaine hydrochloride, PRE-084, DTG, BD 1063, DM and BD 1047 were purchased from Sigma Aldrich (Sigma Aldrich, St. Louis, MO). Gö6976, CTX (Cholera toxin), NF 023, NF 449 and NE-100 were purchased from Calbiochem (Calbiochem, Germany). (+)-SKF 10047, pertussis toxin (PTX) were purchased from Tocris (Tocris, UK).

H-89 and PKAI are protein kinase A (PKA) inhibitors. Gö6976 and BIM I are protein kinase C (PKC) inhibitors. GTPγS is a G protein activator. NF 023 and PTX are G_i/o_ antagonists. NF 449 is a G_s_ antagonist, and CTX is a G_s_ activator. BD 1063, BD 1047 and NE-100 are selective sigma-1 receptor antagonists. DTG, PRE-084, (+)-SKF 10047 and DM are selective sigma-1 receptor agonists.

### Molecular biology

Site-directed F1764A mutagenesis was achieved in the Na_V_1.2 Na^+^ channel clone (the rat brain type IIA Na^+^ channel clone kindly provided by Alan L. Goldin [22]) using the KOD-Plus mutagenesis system (TOYOBO, Japan). The Na_V_1.2 mutant Y1771A was kindly provided by Professor William A. Catterall [23]. The Na_V_1.4 Na^+^ channel clone was incorporated into the pEGFP-N1 in our lab using the rat Na_V_1.4 gene mRNA sequence (NM013178) on the NCBI website. Site-directed F1579A and Y1586A mutageneses were achieved in the Na_V_1.4 Na^+^ channel using the KOD-Plus mutagenesis system (TOYOBO, Japan). The homo sigma-1R gene (NM_005866) with a flag tag was incorporated into a pCDNA3 vector. The siRNA sequence that corresponded to nucleotides 500–519 of the human sigma-1 receptor open-reading frame (NM005866) was inserted into a pGPU6/GFP/Neo plasmid (GenePharm, Shanghai) to generate vector-based siRNA. All of these constructs were confirmed by sequencing.

SYBR Green-based Real-time RT-PCR was conducted to detect mRNA expression of sigma-1 receptors in COS-7 cells. The primers for sigma-1 receptor: forward primer: 5′ – GCTGCAGTGGGTGTTCGTGAATG -3′ and reverse primer: 5′ – GGTGGAAGGTGCCAGAGATGATGGTA -3′. The mRNA expression of sigma-1 receptors was normalized by the housekeeping gene GAPDH (forward primer: 5′- GAGTCAACGGATTTGGTCGT -3′ ; reverse primer: 5′- AATGAAGGGGTCATTGATGG -3′). The PCR conditions were as follows: 94°C, 5 min; 38 cycles of 94°C, 30 s; 58°C, 30 s; 72°C,30 s; 72°C, 8 min. Cycle threshold (Ct) values and concentrations of samples were calculated using Bio-RAD iCycler software.

### Cell culture and transfection

HEK293T cells and COS-7 cells were purchased from the cell bank of Chinese Academy of Sciences (Shanghai, China). Both cell lines were grown in Dulbecco's modified Eagle's medium (DMEM, GIBICO) supplemented with 10% fetal bovine serum and a 1% antibiotic antimycotic solution in 35-mm Petri dishes (Corning Life Sciences, Lowell, MA). Transient transfections with the Na_V_1.2, Na_V_1.4, Na_V_1.5, flag-sigma-1 receptor, sigma-1 receptor RNAi plasmid and mutant channels were performed using Lipofectamine 2000 reagents (Invitrogen, USA) according to the manufacturer's instructions. The cells were used for patching or other biochemical tests 48 h after transfection.

Cerebellar granule neurons were derived from cerebellum of 7-day-old Sprague–Dawley rat pups as described previously [24]. Isolated cells were then plated onto 35-mm-diameter Petri dishes coated with poly-l-lysine (1 µg/mL) at a density of 2.5×10^5^/cm^2^. Cultured cells were incubated at 37°C with 5% CO_2_ in Dulbecco's Modefied Eagle's medium supplemented with 10% fetal calf serum, glutamine (5 mm), insulin (5 µg/mL), KCl (25 mm), and 1% antibiotic/antimycotic solution. All experiments were carried using cerebellar granule neurons at 7–9 days in culture.

### Western blot

Cell homogenates were prepared using HEPES-NP40 lysis buffer (20 mM HEPES, 150 mM NaCl, 0.5% NP-40, 10% glycerol, 2 mM EDTA, 100 µM Na_3_VO_4_, 50 µM NaF, pH 7.5). The protein samples were resolved using 10% SDS PAGE and transferred to polyvinyldifluoride (PVDF) membranes (Millipore, 0.45 µm) in a transfer buffer (25 mM Tris, 192 mM glycine, 20% methanol, v/v) at 100 V for 1 h. The PVDF membranes were blocked with 10% nonfat dry milk in TBST (TBS containing 0.05% Tween 20) for 1 h at room temperature. The membranes were incubated with the primary antibody, anti-sigma-1 receptor [Rabbit polyclonal antibody (a gift from Dr. Teruo Hayashi, Japan) in TBST with 5% BSA (bovine serum albumin)], overnight at 4°C. The blot was washed 3 times for 10 min in TBST and incubated with a horseradish peroxidase-conjugated secondary antibody (1/20,000) (KangChen Bio-tech) in TBST with 5% nonfat dry milk for 1 h at room temperature. The blots were developed using enhanced chemiluminescence (ECL) reagents from Pierce and the ChemiDoc XRS+imaging system from Bio-Rad.

### Electrophysiology

Whole-cell currents in the HEK293T and COS-7 cells were recorded using an Axopatch 200B amplifier (Axon Instruments, Sunnyvale, CA). The bath solution contained (in mM) 145 NaCl, 2.5 KCl, 10 HEPES and 1 MgCl_2_ (pH adjusted to 7.4 using NaOH). The internal solution contained (in mM) 140 CsCl, 4 KCl, 10 HEPES, and 5 EGTA (pH adjusted to 7.4 using CsOH). The pipettes were created from capillary tubing(BRAND, Wertheim, Germany) and had resistances of 5 to 7 MΩ under these solution conditions. All of the recordings were performed at room temperature. A superimposed Na^+^ current was evoked by a 30-ms depolarizing pulse from a holding potential of −100 to −20 mV. Steady-state Na^+^ channel activation was obtained using the following protocol. The cells were held at −100 mV and depolarized in 10-mV steps from −70 to +30 mV at 10-s intervals. The normalized conductance was plotted as a function of the command potential. The conductance was calculated as *G*_Na_ = *I*_Na_/(*V*m-*V*rev). The data points were fitted using the Boltzmann function: *G*_Na_/*G*_Na-max_ = 1/{1+exp[(*V*_m1/2_-*V*_m_)/k]}. Steady-state inactivation of Na^+^ channel was achieved using the following protocol. Five hundred millisecond conditioning pre-pulses ranging from −130 to −20 mV in 10-mV increments were applied prior to the −20 mV test pulse. The peak current amplitudes were normalized to the maximum current and plotted against the pre-pulse potential. The normalized current points were fitted using the Boltzmann function: I_Na_/I_Na-max_ = 1/{1+exp[(*V*_m_-*V*_m1/2_)/k]}+A. The currents were sampled at 10 kHz and filtered at 3 kHz. The currents were corrected online for leak and residual capacitance transients using a P/4 protocol.

### Data acquisition and analysis

The data acquisition and analysis were performed with pClamp 8.01 (Axon Instruments) and/or Origin 7.5 software (MicroCal, Northampton, MA). The statistical analysis consisted of unpaired or paired (depending on the circumstances) Student's T tests. Values are given as the means±SEM, and n indicates the number of tested cells. P<0.05 was defined to be a statistically significant difference between groups. Multiple comparisons were analyzed using a one-way analysis of variance (ANOVA) followed by the post-hoc Tukey test. The dose-response curve was fitted by a sigmoidal dose-response equation: I/I_max_ = 1/(1+10 ∧ (log IC50 – [SKF]) *p), where IC_50_ is the concentration producing half maximal block, [SKF] is the SKF 10047 concentration, p is the Hill coefficient.

## Results

### (+)-SKF 10047 inhibits Na_V_1.2 channel currents in a dose-dependent manner

**Figure 1:**
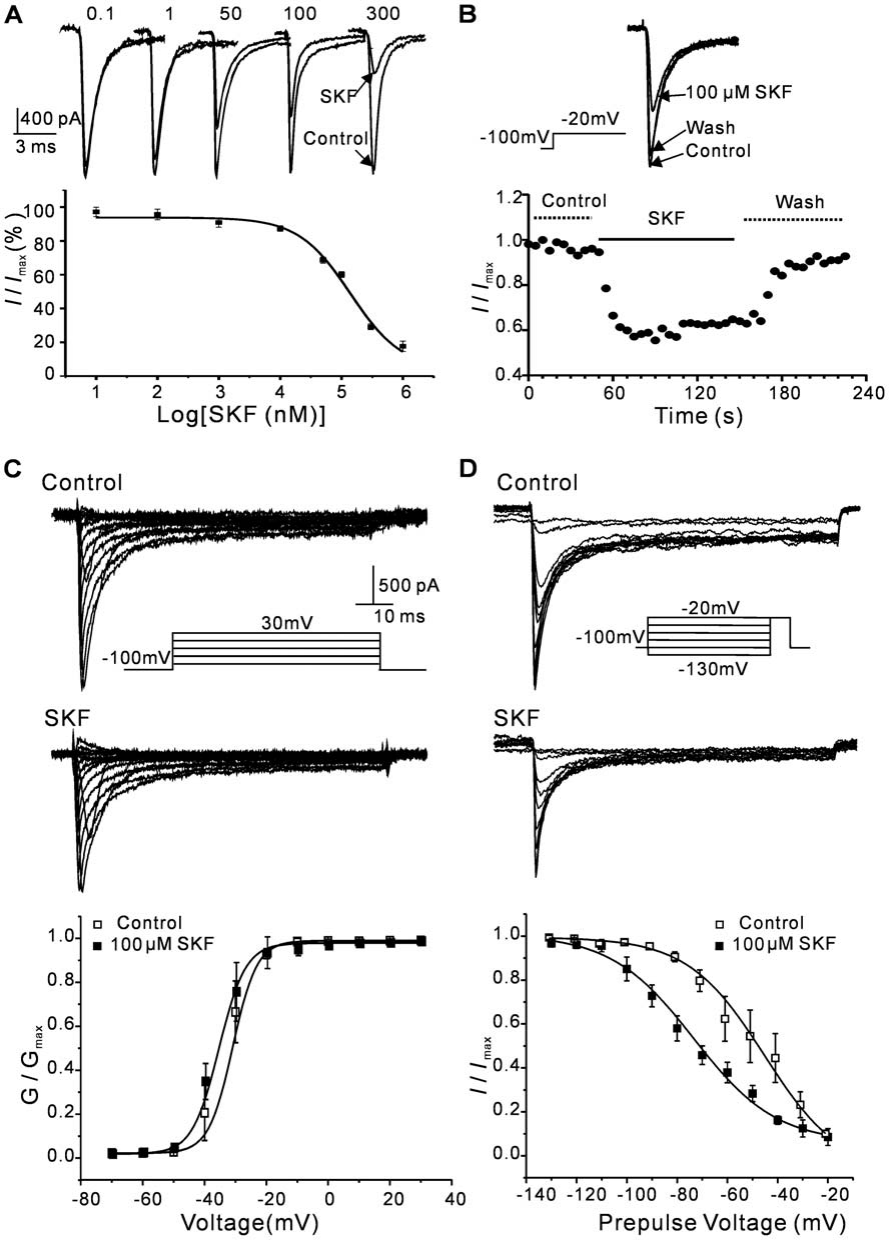
(+)-SKF 10047 inhibition of the Na_V_1.2 current in transfected HEK293T cells. A, representative current traces of Na_V_1.2 current blocked by various concentrations of (+)-SKF 10047 (0.1∼300 µM, top). The currents were elicited by a test pulse of −20 mV from a holding potential of −100 mV. The dose-response curve was fitted using the equation given in methods. The half-maximal inhibition value was 140 µM and the Hill coefficient was −1.0. (bottom). B, A sample current shows the reversible 100 µM (+)-SKF 10047 inhibition of the Na_V_1.2 current (top). The time course of the Na_V_1.2 current inhibition by 100 µM (+)-SKF 10047 (bottom). C, The effect of (+)-SKF 10047 on steady-state activation of Na_V_1.2 . The current traces show the voltage-dependent Na_V_1.2 current activation curves in the absence (top) and presence (middle) of (+)-SKF 10047; the normalized data points were fitted using the Boltzmann equation (bottom). (+)-SKF 10047 did not alter the steady-state Na_V_1.2 activation. P>0.05, n = 5. D, The effect of (+)-SKF 10047 on steady-state inactivation of Na_V_1.2. The control currents (top) and currents following treatment with 100 µM (+)-SKF 10047 (middle) are shown. The normalized data points were fitted using the Boltzmann function (bottom).

*I*_Na_ currents were elicited by a 30-ms depolarizing pulse to −20 mV from a holding potential of −100 mV at 10 s intervals. The currents were recorded for 1 min to obtain a stable baseline during blank solution perfusion, and the drug solution was perfused until a stable inhibition level was achieved. (+)-SKF 10047 inhibited the Na_V_1.2 channel currents in a dose-dependent manner (0.01 µM: 2.8±2.7%, n = 5; 0.1 µM: 5.7±2.1%, n = 5; 1 µM: 12.7±1.5%, n = 6; 50 µM: 31±1.5%, n = 6; 100 µM: 39.5±1.3%, n = 6; 300 µM: 71±1.7%, n = 6; 1000 µM : 82.4±3.1%, n = 6; P<0.05, Fig 1A). The dose-response curve was fitted using equation given in the method, and the IC_50_ value and Hill coefficient are 140 µM and −1.0 respectively (Fig1 A). The inhibitory effect of (+)-SKF 10047 on *I*_Na_ began quickly and reached a maximum effect within 90 s. The inhibition was reversible within 1–2 min (Fig 1B).

The effects of (+)-SKF 10047 on steady-state Na_V_1.2 channel inactivation and activation were tested using 100 µM SKF10047. The steady-state activation was determined using a 70-ms depolarizing pulse from a holding potential of −100 mV to potentials between −70 and +60 mV in 10-mV steps at 10-s intervals. The (+)-SKF 10047 did not significantly alter the voltage-dependence of the Na_V_1.2 channel steady-state activation (control: *V*_1/2_ = −32.9±2.6 mV, n = 5; (+)-SKF 10047: *V*_1/2_ = −33.4±1.4 mV, n = 12; P>0.05, Fig 1C). The steady-state inactivation was examined using a −20 mV test depolarization from the holding potential in 10-mV increments (−130 mV to −20 mV; 2 s). (+)-SKF 10047 significantly shifted the steady-state Na_V_1.2 inactivation by approximately −27 mV towards negative potentials (control: *V*_1/2_ = −50.1±8.0 mV, n = 5; SKF: *V*_1/2_ = −77.1±3.9 mV, n = 5; P<0.05, Fig 1D).

### (+)-SKF 10047 inhibits Na_V_1.2/1.4 channel currents through a sigma-1 receptor-independent pathway

**Figure 2:**
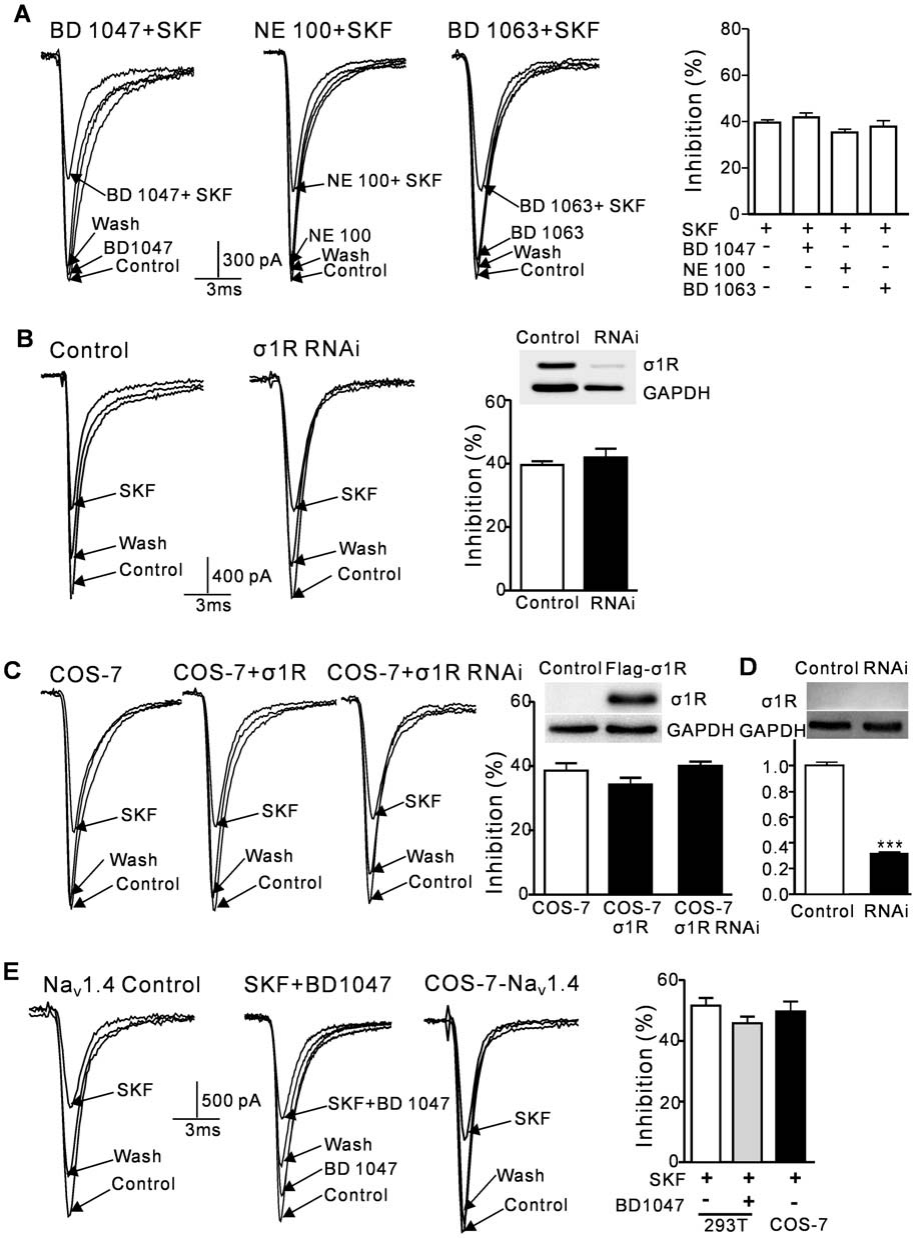
The inhibitory effects of (+)-SKF 10047 on Na_V_1.2 and Na_V_1.4 are independent of sigma-1 receptor activation. A, The sample currents for BD 1047, NE-100 and BD 1063 inhibition of Na_V_1.2 in HEK293T cells with and without (+)-SKF 10047. The sigma-1 receptor antagonists BD 1047, NE-100 and BD 1063 did not prevent (+)-SKF 10047 -evoked inhibition of Na_V_1.2 in the HEK293T cells (right). B, The effect of (+)-SKF 10047 on Na_V_1.2 in the HEK293T cells was not reduced by sigma-1 receptor shRNA plasmid coexpression. The sigma-1 receptor protein levels were reduced by 58.7% in the Western blot. C, SKF10047 inhibition of Na_V_1.2 currents was similar in the COS-7 cells with/without sigma-1 receptor overexpression and knockdown. The Western blot of the sigma-1 receptor expression level (top right). D, The protein expression of sigma-1 receptors was hardly detectable in the Western blot (top). The normalized mRNA expression of sigma-1 receptors in COS-7 cells, and the sigma-1 receptor mRNA expression was reduced by 70% (bottom). E, The *I*_Na_ for Na_V_1.4 in the HEK293T cells was also reversibly inhibited by SKF, and it was not blocked by BD 1047. The SKF10047 inhibition of Na_V_1.4 channel currents was similar in the COS-7 and HEK293T cells.

We performed three different experiments to investigate (+)-SKF 10047 inhibition of Na_V_1.2 channels by sigma-1 receptor activation. First, the selective sigma-1 receptor antagonists BD 1047, NE-100 and BD 1063 were used to block the inhibitory effect of (+)-SKF-10047. The effects of (+)-SKF 10047 (100 µM) on *I*_Na_ were not altered in the presence of BD 1047 (2 µM), NE-100 (5 µM) or BD 1063 (2 µM) (Fig 2A). The average (+)-SKF 10047 *I*_Na_ inhibitions were 41.8±2.0% (n = 6) , 35.2±1.6% (n = 5), 37.8±2.6% (n = 5) in the presence of BD 1047, NE-100 and BD 1063, respectively, which were not significantly different from the effect of (+)-SKF 10047 alone (39.5±1.3%, n = 6; P>0.05, Fig 2A). Second, the siRNA technique was used to block the inhibitory effect of (+)-SKF-10047. A sigma-1 receptor siRNA construct was designed to target nucleotides 500–519 in the human sigma-1 receptor gene, as has been previously reported [21]. The sigma-1 receptor protein expression in the HEK293T cells was reduced by 58.7% after transfection with this siRNA construct (Fig 2B). The inhibitory effects of (+)-SKF 10047 on the HEK293T cells with the control vector and the HEK293T cells with the sigma-1 receptor RNAi vector were similar (control: 39.5±1.25%, n = 6; RNAi: 41.9±2.8%, n = 5; P>0.05, Fig 2B). The sigma-1 receptor was barely expressed in the COS-7 cells (Fig 2C, D), which is consistent with previous studies [21], [25]. Third, the inhibitory effect of (+)-SKF 10047 on COS-7 cells was investigated. No significant differences between the HEK293T and COS-7 cells were observed (HEK293T: 39.5±1.3%; COS-7: 38.5±2.4%, n = 6; P>0.05). Sigma-1 receptor overexpression in the COS-7 cells did not alter the inhibitory effect of (+)-SKF 10047 on *I*_Na_ (COS-7: 38.5±2.3%, n = 6; COS-7 with sigma-1 receptor overexpression: 33.1±2.8%, n = 7; P>0.05, Fig 2C). In order to further confirm that (+)-SKF 10047 inhibition of Na_V_1.2 currents is independent of sigma-1 receptor activation, we also carried out sigma-1 receptor RNAi experiment in COS-7 cells. Because the sigma-1 receptor expression was hardly detectable by western blot, the quantitative real-time PCR was used to measure the mRNA expression level (Fig2D). The mRNA expression level was reduced 70% by RNAi. Knockdown of the sigma-1 receptor expression in the COS-7 cells did not alter the inhibitory effect of (+)-SKF 10047 on *I*_Na_ (COS-7: 38.5±2.3%, n = 6; COS-7 with sigma-1 receptors RNAi: 41.2±1.4%, n = 6; P>0.05, Fig 2C).

The impact of (+)-SKF 10047 on other Na^+^ channels was also investigated. The inhibitory effect of (+)-SKF 10047 on Na_V_1.4 was examined in the HEK293T and COS-7 cells. No significant differences between the HEK293T cells transfected with Na_V_1.4 (51.5±2.5%, n = 5) and the COS-7 cells transfected with Na_V_1.4 (49.9±3.3% n = 5; P>0.05) were observed. The sigma-1 receptor antagonist BD 1047 was used to investigate the role of sigma-1 receptor activation in (+)-SKF 10047 inhibition of Na_V_1.4. BD 1047 did not alter the (+)-SKF 10047-evoked inhibition of Na_V_1.4 (47.7±2.2%, n = 4, P>0.05 compared to (+)-SKF 10047 alone).

### (+)-SKF 10047 inhibition of Na_V_1.2 currents is independent of the PKA, PKC, and G protein pathway

**Figure 3:**
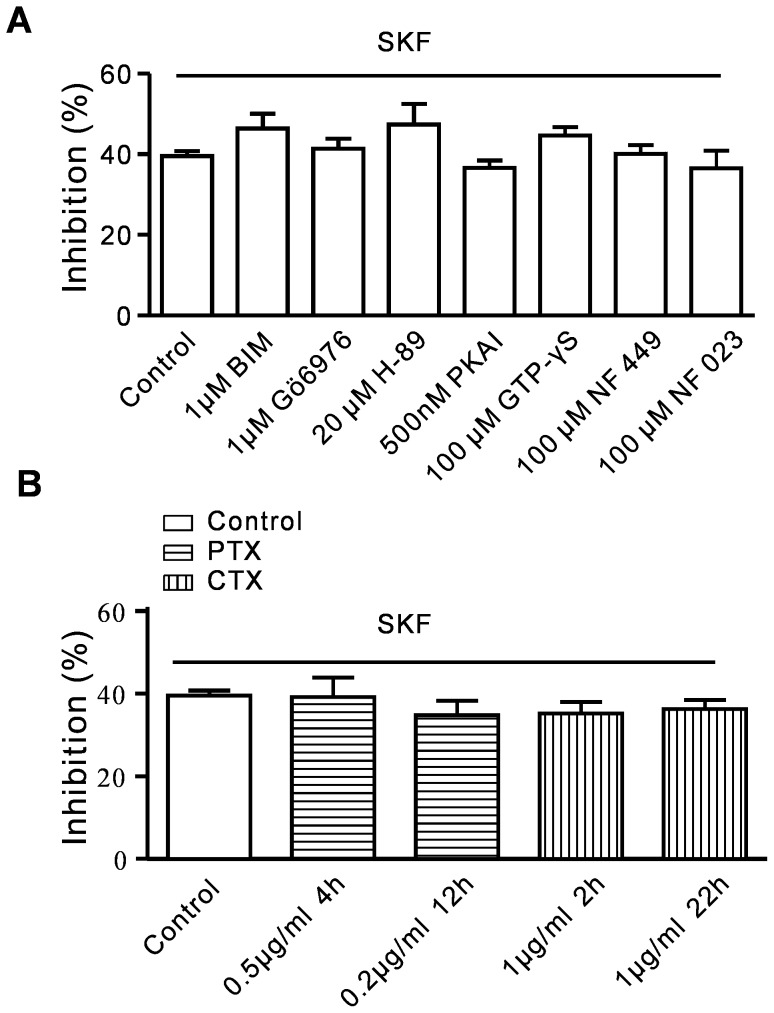
Effect of (+)-SKF 10047 on Na_V_1.2 in the presence of G protein and PKC or PKA signaling pathway inhibitors. A, Histograms of the means±SEM of the peak current inhibition percentages recorded from the Na_V_1.2-transfected HEK293T cells. The inhibitory effect of (+)-SKF 10047 on Na_V_1.2 was not altered by the presence of PKA inhibitors (H-89 and PKAi), PKC inhibitors (BIM I and Gö6976), a G protein agonist (GTPγS), a G_s_ antagonist (NF 449) or a G_i/o_ antagonist (NF 023) in the pipette solution. P>0.05, compared to SKF-1007 alone. B, Pre-incubating the HEK293T cells with CTX (a G_s_ activator) or PTX (a G_i/o_ inhibitor) for different lengths of time and at different concentrations did not alter the inhibitory effects of (+)-SKF 10047 on Na_V_1.2. (P>0.05, compared to the untreated cells).

The data above suggested that (+)-SKF 10047 inhibited Na_V_1.2 currents either directly or via signaling pathways that were independent of sigma-1 receptors. Na_V_1.2 is inhibited by G-protein, PKC and PKA pathway activation [26]. Therefore, we investigated the roles of the PKA, PKC and G-protein pathways in (+)-SKF-10047-evoked inhibition of Na_V_1.2 currents. When the PKA inhibitors H-89 (20 µM) and PKAI (500 nM) were present in the internal solution, (+)-SKF 10047 inhibited *I*_Na_ by 47.4±5.0% (n = 5) and 36.6±1.8% (n = 6), respectively. However, this inhibition was not significantly different with that of 100 µM (+)-SKF 10047 alone (39.5±1.3, n = 6; P>0.05) (Fig 3A). The (+)-SKF 10047 inhibitory effects in the presence of the PKC inhibitors BIM I (1 µM) and Gö6976 (1 µM) were not significantly different from that of 100 µM (+)-SKF 10047 alone (BIM I: 46.4±3.6%, n = 5; Gö6976: 41.3±2.5%, n = 5; P>0.05, Fig 3A).

The following experiments were performed to investigate the role of G proteins in (+)-SKF 10047 inhibition of Na_V_1.2 channel currents. CTX (a G_s_ activator) and PTX (a G_i/o_ inhibitor) were added to the culture medium at different concentrations and for various lengths of time. The cells were incubated with 1 µg/ml CTX for 2 and 22 h and with 200 ng/ml or 500 ng/ml PTX for 4 or 12 h, respectively. The inhibitory effects of 100 µM (+)-SKF 10047 on Na_V_1.2 channel currents were not altered in the presence of these toxins. The average *I*_Na_ inhibitions were 35.2±2.8% (1 µg/ml CTX for 2 h, n = 5), 36.2±2.3% (1 µg/ml CTX for 22 h, n = 5), 39.2±4.8% (200 ng/ml PTX for 4 h, n = 5) and 34.8±3.4% (500 ng/ml PTX for 12 h, n = 5), which were not significantly different with effect of 100 µM (+)-SKF 10047 alone (P>0.05) (Fig 3B). The inhibitory effect of (+)-SKF 10047 was also not significantly altered by the presence of GTPγS (a G-protein activator), NF 023 (a G_i/o_ antagonist) or NF 449 (a G_s_ antagonist) in the internal solution (Fig 3A).

These results suggested that (+)-SKF 10047 directly inhibited the Na_V_1.2 channel currents.

Therefore, we investigated the frequency- and use-dependency of (+)-SKF 10047 Na_V_1.2 channel current inhibition.

### (+)-SKF 10047 inhibits Na_V_1.2 channel currents in a frequency- and use-dependent manner

**Figure 4:**
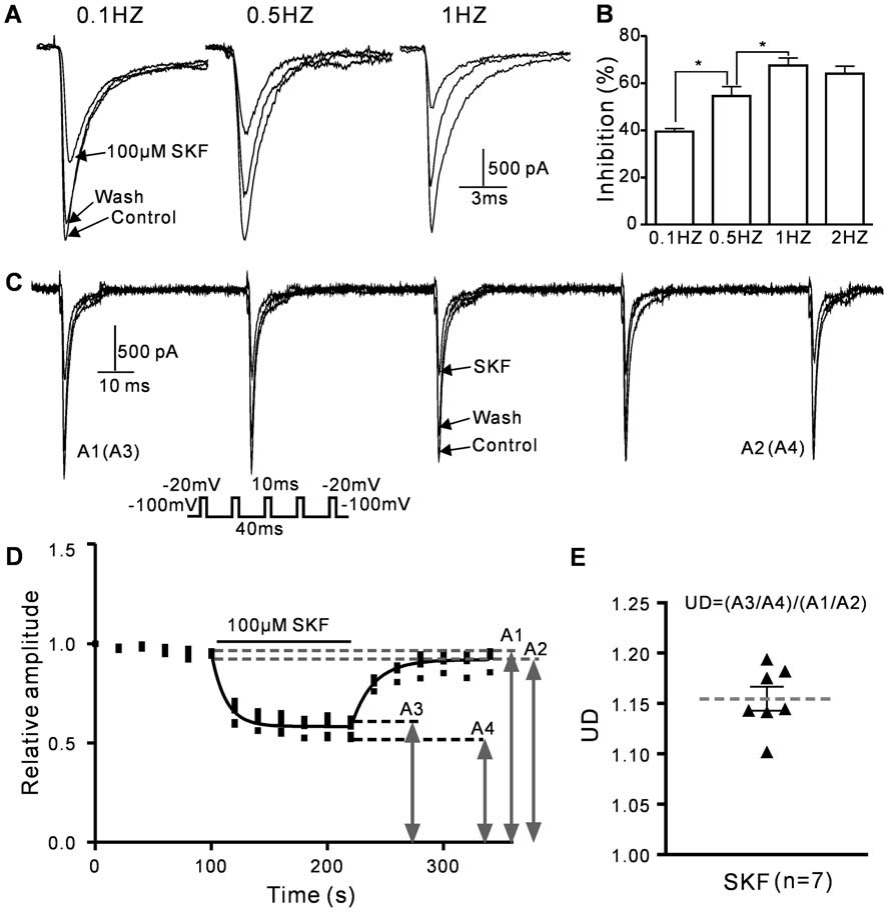
Frequency- and use-dependent inhibitory effect of (+)-SKF 10047 on *I*_Na_. A, Sample current traces at 0.1, 0.5 and 1 Hz pulse frequencies were obtained in the presence and absence of 100 µM (+)-SKF-10047. B, SKF inhibition of *I*_Na_ increased with increased pulse frequency. *, P<0.05 compared to 0.1 Hz. C, Sample Na_V_1.2 current traces for 5 Hz-train depolarizations from −100 to −20 mV were obtained in the presence and absence of 100 µM (+)-SKF-10047. D, The time course of the peak amplitude changes during the train pulses. Use-dependence (UD) = (A3/A4)/(A1/A2). E, The individual UD data points (black triangles) and the UD means±SEM (gray dashed line) under the 5-Hz train pulse condition. The UD ranged from 1.10 to 1.19.

The effect of frequency on (+)-SKF 10047 inhibition of Na_V_1.2 channel currents was investigated using different frequencies of 30-ms pulses that depolarized the holding potential from −100 to −20 mV. The cells were stimulated for 2 min in the continued presence of 100 µM (+)-SKF-10047. The average Na_V_1.2 channel current inhibitions for stimulations at 0.1, 0.5, 1 and 2 HZ were 39.5±1.3% (n = 6), 54.6±4.0% (n = 7), 67.6±3.2% (n = 9), and 64.1±3.2% (n = 7),respectively (P<0.05 compared to 0.1 Hz) (Figs 4A and B). These results suggested that (+)-SKF 10047 exerted frequency-dependent effects on the Na_V_1.2 channel. The use-dependency of the inhibition was evaluated using 5-Hz trains of five pulses that depolarizing the holding potentials from −100 to −10 mV (Fig 4C). The trains were repeated every 20 s. (+)-SKF 10047 (100 µM) was applied after 6 control trains and washed out, and the 6 trains were repeated (Fig 4D). The use-dependence (UD) was determined by the peak amplitudes of the first and last evoked currents of the last train in the presence and absence of (+)-SKF 10047 (UD = (A3/A4)/(A1/A2), Fig 4E). The UD of 7 samples ranged from 1.10 to 1.19 (Fig 4E). These results suggested that the inhibitory effect of (+)-SKF 10047 on the Na_V_1.2 channel was use-dependent and that the drug may preferentially bind to the depolarized (i.e., open or inactivated) channel.

### The effect of (+)-SKF 10047 on the Na_V_1.2 channel is altered by the site-directed mutation of F1764A and Y1771A

**Figure 5:**
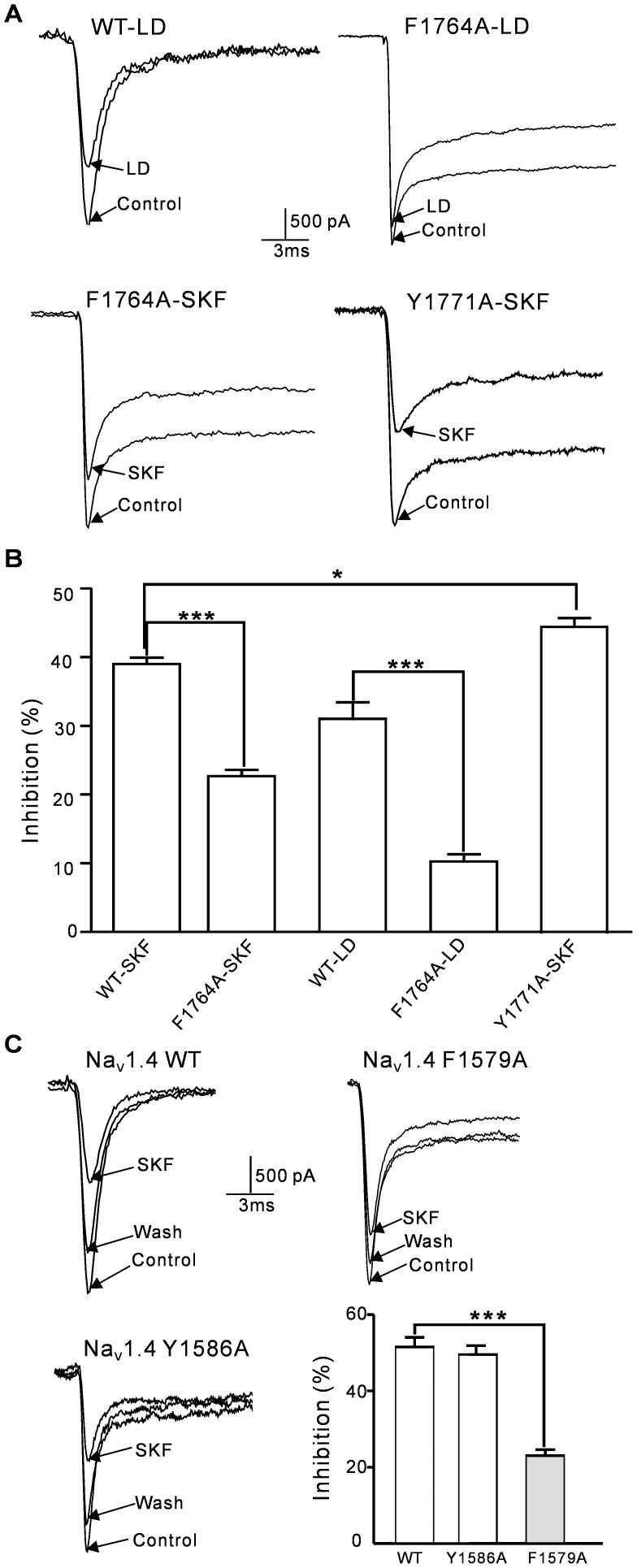
The effects of (+)-SKF 10047 or lidocaine on wild-type and mutant Na_V_1.2/Na_V_1.4 in HEK293T cells. A, Sample current traces for wild-type and mutant Na_V_1.2 channels (F1764A or Y1771A) were obtained in the presence and absence of 100 µM (+)-SKF 10047 or 100 µM lidocaine. B, The statistical analyses of *I*_Na_ inhibition for wild-type and mutant Na_V_1.2 channels in the presence of 100 µM (+)-SKF 10047 or 100 µM lidocaine. The (+)-SKF 10047 inhibition of the F1764A and Y1771A mutant channel currents was significantly different from the inhibition of the wild-type Na_V_1.2 current (*, P<0.05; ***, P<0.001). The lidocaine-induced *I*_Na_ inhibition also differed significantly between the F1764A and wild-type Na_V_1.2 channels (***, P<0.001, Student's t test). C, Sample current traces for wild-type, F1579A and Y1586A mutant Na_V_1.4 channels were obtained in the presence and absence of 100 µM (+)-SKF 10047. The mutation of F1579A significantly reduced the (+)-SKF 10047 inhibition of Na_V_1.4 channel currents (***, P<0.001).

Local anesthetic (LA) drugs, such as lidocaine (LD), may directly inhibit rat Na_V_1.2 channel currents, and the amino acid residues Phe-1764 and Tyr-1771 in the S6 transmembrane segment of domain IV are critical for this inhibition [23], [27]. LD induced less inhibition in the F1764A mutant Na_V_1.2 channel than in the wild-type channel (Figs 5A and B).

F1764A and Y1771A single-point mutant Na_V_1.2 channels were investigated in HEK293T cells to assess similarities in the effects of (+)-SKF 10047 and LD on the Na_V_1.2 channel. The (+)-SKF 10047 inhibition of the F1764A mutant channel currents was significantly less than that of the wild-type channel currents (WT: 39.5±1.3%; F1764A mutant: 25.9±1.6%, n = 9; p<0.001, Fig 5B). Interestingly, the (+)-SKF 10047 inhibitory effect on the Y1771A mutant channel currents was significantly higher than that of the wild-type channel currents (Y1771A mutant: 45.2±1.3%, n = 6, P<0.05 compared to WT). These data suggested the importance of the F1764 and Y1771 residues for (+)-SKF 10047 inhibition of Na_V_1.2 channel currents.

### The effect of (+)-SKF 10047 on the Na_V_1.4 channel is altered by the site-directed mutation of F1579A

The mutations of F1579A and Y1586A, which correspond to the F1764A and Y1771A in Na_V_1.2 channel, were introduced into Na_V_1.4 channels. F1579A and Y1586A single-point mutant NaV1.4 channels were investigated in HEK293T cells. The (+)-SKF 10047 inhibition of the F1579A mutant Na_V_1.4 channel currents was significantly less than that of the wild-type channel currents (WT: 51.5±2.5%, n = 5; F1579A mutant: 23.0±1.7%, n = 7; p<0.001, Fig 5C). The (+)-SKF 10047 inhibition of Na_V_1.4 channel currents was not changed by Y1586A mutation. (WT: 51.5±2.5%, n = 5; Y1586A mutant: 49.3±0.5%, n = 14; p>0.05, Fig 5C).

### The sigma-1 receptor-selective agonists DM and DTG also reduce rat Na_V_1.2 currents

**Figure 6:**
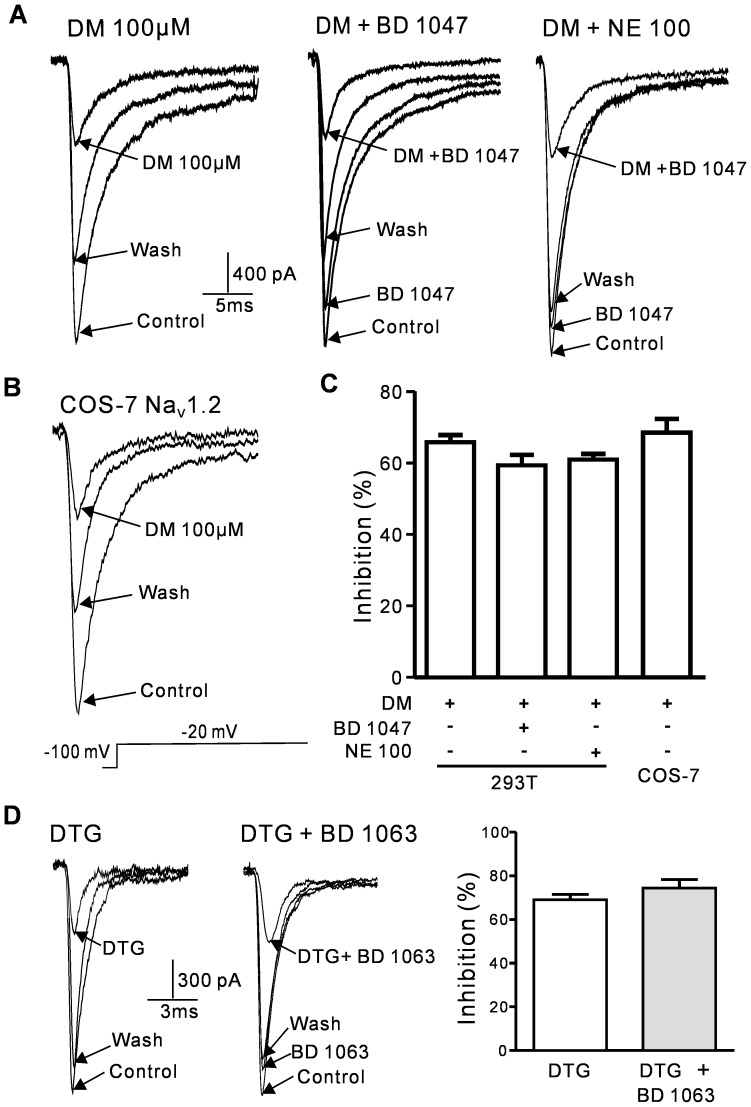
DM and DTG inhibition of Na_V_1.2 channel currents in HEK293T cells was independent of sigma-1 receptor activation. A, Sample current traces showing DM inhibition of Na_V_1.2 channels in the HEK293T cells with and without 2 µM BD 1047 or NE-100. B, Sample current traces showing DM inhibition of Na_V_1.2 channels in the COS-7 cells. C, Histograms of the means±SEM of the peak current inhibition percentages of DM. The *I*_Na_ inhibitory effects of DM+2 µM BD 1047 and DM+2.5 µM NE-100 were similar to the effects of 100 µM DM. The DM inhibition of *I*_Na_ was not significantly different between the HEK293T and COS-7 cells. D, Sample current traces showing DTG inhibition of Na_V_1.2 channels in the HEK293T cells with and without 2 µM BD 1063. The average percent inhibition of *I*_Na_ by DTG in the absence and presence of BD 1063 (right).

The sigma-1 receptor-selective agonists DM, DTG and PRE-084 were used to assess the inhibitory effects of other sigma-1 receptor ligands on rat Na_V_1.2 channels. PRE-084 exerted little effect on the Na_V_1.2 channel currents (data not shown), but DM and DTG reversibly inhibited the Na_V_1.2 channel currents (Fig 6A, D). The average inhibition from 100 µM DM in the Na_V_1.2-transfected HEK293T cells was 65.8±2.0% (n = 6). The sigma-1 receptor antagonists BD 1047 and NE-100 were used to investigate the role of sigma-1 receptors in this inhibitory effect. The average DM inhibitions in the presence of 2 µM BD 1047 and 2.5 µM NE-100 were 59.7±2.9% (n = 5) and 61.2±1.6% (n = 5), respectively. These results were not significantly different from those observed with DM alone (Fig 6C). DM inhibition of Na_V_1.2 channel currents was also investigated in the COS-7 cells. The inhibitory effect of DM on Na_V_1.2 channel currents in the COS-7 cells was similar to the effect in the HEK293T cells (COS-7: 68.7±3.9%, n = 5; HEK293T: 65.8±2.0%, n = 6; P>0.05; Figs 6B and C). Previous study has been shown that DTG effect (via activation of the sigma-1 receptor) could be blocked by BD 1063 [28]. Our results showed that The DTG inhibition of Na_V_1.2 channel currents was not altered by sigma-1 receptor antagonist BD 1063 (100 µM DTG: 69.0±2.5%, n = 6; 100 µM DTG+2 µM BD 1063: 71.8±3.6%, n = 4; P>0.05).These results suggested that DM and DTG inhibited Na_V_1.2 currents via a sigma-1 receptor-independent pathway.

### (+)-SKF 10047 inhibits sodium currents in rat cerebellar granule neurons

**Figure 7:**
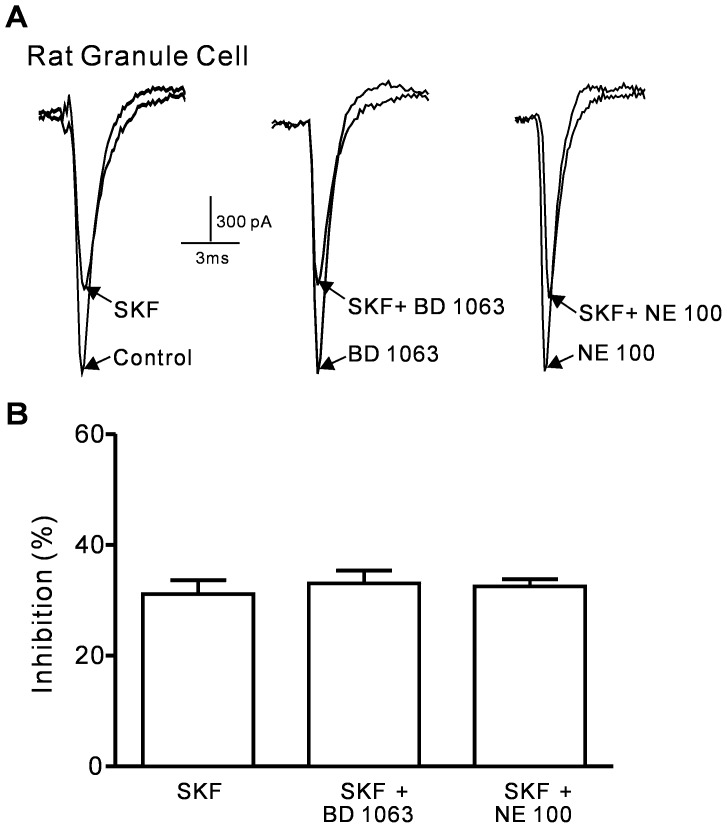
(+)-SKF 10047 inhibition of *I*_Na_ in rat cerebellar granule cells was not affected by sigma-1 receptor antagonist. A, Sample current traces showing SKF 10047 inhibition of *I*_Na_ in rat cerebellar granule cells with and without 2 µM BD 1063 and 2.5 µM NE-100. B, Histograms of the means±SEM of the peak current inhibition percentages of SKF 10047. The *I*Na inhibitory effect of SKF 10047 was not altered by BD 1063 or NE-100.

The primary cultured rat cerebellar granule cells, which express mainly Na_V_1.2 and Na_V_1.6 channel [29], were used to test whether (+)-SKF 10047 has similar inhibitory effect on native sodium currents. 100 µM (+)-SKF 10047 reversibly inhibited sodium currents in granule cells and the inhibition was not blocked by BD 1063 and NE-100 (SKF: 31.1±2.6%, n = 4; SKF+BD 1063: 33.1±2.3%, n = 5; SKF+NE-100: 32.5±1.3%, n = 4; P>0.05, Fig 7A,B).

## Discussion

(+)-SKF 10047 is a prototypic and specific sigma-1 receptor agonist that has been extensively used to study sigma-1 receptor function. By activation of sigma-1 receptors, (+)-SKF 10047 inhibits N-methyl-D-aspartate (NMDA) receptors in rat retinal ganglion cells [30], voltage-gated cardiac Na_V_1.5 channels [20], [21], [25], L-type voltage-gated calcium channels [31], various types of voltage-gated K^+^ channels [32], [33], [34]. (+)-SKF 10047 also inhibits glutamate release in rat cerebral cortex neurons [35]. We found that (+)-SKF 10047 inhibited Na_V_1.2 and Na_V_1.4 voltage-gated sodium channels, and this inhibitory effect was not eliminated by the sigma-1 selective antagonists BD 1047 and NE-100. These results suggest that (+)-SKF 10047 inhibition of Na_V_1.2/1.4 channel currents is independent of sigma-1 receptors.

Sigma-1 receptor ligands likely exert effects that are unrelated to sigma-1 receptors. NDHEA sulfate, a sigma-1 receptor agonist, inhibits persistent sodium currents in the rat medial prefrontal cortex via the G_i_ protein and the PKC signaling pathways [36]. The sigma-1 receptor ligand PRE-084 amplifies dopamine D1 receptor signaling in the prelimbic cortex [37]. G-protein blockade or activation using G_i/o_ and G_s_ inhibitors did not alter the (+)-SKF 10047 -induced inhibition of Na_V_1.2 channel currents. The inhibitory effect of (+)-SKF 10047 on Na_V_1.2 channel currents persisted in the presence of PKA and PKC inhibitors (Fig3). These results further suggest that (+)-SKF 10047 inhibition of Na_V_1.2 channel currents is independent of sigma-1 receptors, G-protein-coupled receptor (GPCRs) activity or phosphokinase pathways.

Most local anesthetics are state-dependent blockers of Na^+^ channels. The mechanism of this blockade may be the high affinity of these drugs for a site on the opening, inactivated and resting channel [38], [39]. (+)-SKF 10047 inhibited Na_V_1.2 channels in a state-dependent manner, which is consistent with Na^+^ channel blockade. (+)-SKF 10047 preferentially interacted with inactivated Na_V_1.2 channels to produce a significant hyperpolarizing shift in the voltage-dependent inactivation, which reduced channel availability and slowed recovery. The marked effects of (+)-SKF 10047 on channel inactivation are consistent with the high affinity of local anesthetics and anticonvulsants, such as LD, for inactivated channels [23], [27]. (+)-SKF 10047 also produced a use-dependent blockade of the Na_V_1.2 channel following high-frequency stimulation, which suggested an (+)-SKF 10047 affinity for open or inactivated Na^+^ channels. This result suggests that (+)-SKF 10047 may directly inhibit Na_V_1.2 channels.

The direct interactions between local anesthetics and Na_V_1.2 channels have been thoroughly studied, and Phe-1764 and Tyr-1771 mutations in domain IV of the S6 channel transmembrane segment dramatically reduce Na_V_1.2 channel current inhibition by local anesthetics, such as lidocaine [23], [27]. We found that an F1764A mutation dramatically slowed the fast inactivation of the Na_V_1.2 current, which is consistent with a previous report [27]. This mutation also reduced the (+)-SKF 10047 inhibition of Na_V_1.2 channel currents by 35%. However, the Y1771A mutation did not reduce (+)-SKF 10047 inhibition of Na_V_1.2 channels (Fig 5). These results suggest that the sites or mechanisms of (+)-SKF 10047 binding to Na_V_1.2 channels are not identical to those of lidocaine. Previous studies suggested that F1764 and Y1771 are direct LA (local anesthetic) drugs binding sites of sodium channels [27], [40]. Both F1764A and Y1771A mutations reduced the LA affinity for open and fast inactivated channels, but the effect of Y1771A was much less than F1764A [27]. Our data showed that Y1771A increased the inhibition of SKF10047, which may similar to the mutations I1761A, V1776A and N1769A increasing the LA drugs inhibition of Na_V_1.2 channels [27]. Y1771A may increase closed- state Na_V_1.2 channel sensitivity to SKF 10047 in an indirect way. Since SKF 10047 binds preferentially to inactivated-state channels, Y1771A may partially change the SKF10047 binding site of close-state Na_V_1.2 channels towards the inactivated conformation. The specific structural and mechanistic differences that determine the effects of (+)-SKF 10047 on Na_V_1.2 may involve multiple binding sites. However, this hypothesis requires detailed structure–function investigations. The F1579A mutation (corresponding to F1764 in Na_V_1.2 channel) significantly reduced the (+)-SKF 10047 inhibition of Na_V_1.4 channel currents, but the Y1586A mutation showed no effect. This is consistent with the previous report that the affinities of local anesthetic drug binding to Na_V_1.4 channel are determined primarily by interaction with F1759 [40]. Since it has more restricted conformation than lidocaine and Y1586 is on the bottom of channel pore comparing to F1579, (+)-SKF 10047 may not direct interact with Y1586 site.

Interestingly, (+)-SKF 10047 directly inhibited both Na_V_1.2 and Na_V_1.4 channels. These inhibitory effects occurred and were washed out within 1–2 minutes. The IC_50_ value of Na_V_1.2 for (+)-SKF 10047 was 140 µM. These results are different from previous reports on (+)-SKF 10047 inhibition of cardiac Na_V_1.5 channels via sigma-1 receptor activation, which required more than 10 min to occur and wash out and had a much lower IC_50_ Value (70 µM) [21]. The inhibitory effects of (+)-SKF 10047 on Na_V_1.5 channels also differed between HEK293 cells with abundant sigma-1 receptor expression and COS-7 cells with little sigma-1 receptor expression [21], [25]. This study compared SKF-1047 inhibition of Na_V_1.2 and Na_V_1.4 channels in HEK293T cells and COS-7 cells, and no differences between these two cell types were observed (Fig 2). SKF-10047 directly inhibited the COS-7 cells, which express Na_V_1.2 and Na_V_1.4 channels but few sigma-1 receptors. The Na_V_1.2, Na_V_1.4, and Na_V_1.5 α-subunit isoforms have greater than 60% amino acid sequence identity. However, these channels exhibit gating, permeability, and conductance functional differences, which result in tissue-specific physiological functions and subtle difference in their pharmacological properties [10]. The markedly different (+)-SKF 10047 inhibition of Na_V_1.2/Na_V_1.4 and Na_V_1.5 currents may be explained by chemical structure differences because the Na_V_1.5 protein harbors multiple evolutionary conserved amino acid motifs for N-glycosylation in its extracellular domain. The N-glycosylation affects Na_V_1.5 channel gating [41] , and maybe also affect SKF 10047 binding to the channel.

(+)-SKF 10047 may inhibit various ion channels via sigma-1 receptor activation, but a direct interaction has seldom been noted. Lamy et al. have recently reported that the sigma-1 agonist DTG directly inhibits small-conductance Ca^2+^-activated K channels in dopaminergic neurons and HEK-293 cells [42]. In addition, DM directly inhibits brain Na^+^ channels in Xenopus oocytes [43]. The sigma-1 agonists DTG, (+)-SKF 10047 and DM directly inhibited the Na_V_1.2 and/or Na_V_1.4 channel currents in the present study. DM and (+)-SKF 10047 belong to the homologous family of benzomorphan compounds. Therefore, further investigation of the effects of other benzomorphan compounds is required.

The (+)-SKF 10047 inhibitory effect was also tested in primary cultured rat cerebellar granule neurons. The sigma-1 receptor antagonist BD 1063 and NE-100 failed to block the (+)-SKF 10047 inhibition of sodium currents in the granule neurons, which suggested that the inhibition was independent of the sigma-1 receptor activation. However, rat cerebellar granule neurons express both Na_V_1.2 and Na_V_1.6 channels [29], the (+)-SKF 10047 effect on Na_V_1.6 channel currents need further investigation.

In conclusion, our study found that the sigma-1 receptor agonists DTG, (+)-SKF 10047 and DM directly inhibited Na_V_1.2 or Na_V_1.4 channels in transfected HEK293T and COS-7 cells. The sigma-1 receptor is involved in many diseases, and the final action of sigma-1 receptor activation is most likely to modulate various ion channels. Therefore, the direct effects of sigma-1 receptor ligands on ion channels should receive special attention.
